# Serotonin Differentially Regulates Short- and Long-Term Prediction of Rewards in the Ventral and Dorsal Striatum

**DOI:** 10.1371/journal.pone.0001333

**Published:** 2007-12-19

**Authors:** Saori C. Tanaka, Nicolas Schweighofer, Shuji Asahi, Kazuhiro Shishida, Yasumasa Okamoto, Shigeto Yamawaki, Kenji Doya

**Affiliations:** 1 Department of Computational Neurobiology, ATR Computational Neuroscience Laboratories, Seika, Souraku, Kyoto, Japan; 2 Core Research for Evolutional Science and Technology (CREST), Japan Science and Technology Agency, Seika, Souraku, Kyoto, Japan; 3 Department of Biokinesiology and Physical Therapy, University of Southern California, Los Angeles, California, United States of America; 4 Department of Psychiatry and Neurosciences, Hiroshima University, Minamiku, Hiroshima, Japan; 5 Neural Computational Unit, Okinawa Institute of Science and Technology, Suzaki, Uruma, Okinawa, Japan; National Institutes of Health, United States of America

## Abstract

**Background:**

The ability to select an action by considering both delays and amount of reward outcome is critical for maximizing long-term benefits. Although previous animal experiments on impulsivity have suggested a role of serotonin in behaviors requiring prediction of delayed rewards, the underlying neural mechanism is unclear.

**Methodology/Principal Findings:**

To elucidate the role of serotonin in the evaluation of delayed rewards, we performed a functional brain imaging experiment in which subjects chose small-immediate or large-delayed liquid rewards under dietary regulation of tryptophan, a precursor of serotonin. A model-based analysis revealed that the activity of the ventral part of the striatum was correlated with reward prediction at shorter time scales, and this correlated activity was stronger at low serotonin levels. By contrast, the activity of the dorsal part of the striatum was correlated with reward prediction at longer time scales, and this correlated activity was stronger at high serotonin levels.

**Conclusions/Significance:**

Our results suggest that serotonin controls the time scale of reward prediction by differentially regulating activities within the striatum.

## Introduction

When hungry and looking for a restaurant, do you choose a well-reputed restaurant with many people waiting in line or a fast food restaurant where you can have a quick but perhaps less palatable meal? In our daily life, we constantly make such choices between actions leading to rewards of various sizes after different delays. “Delay discounting” is a theoretical concept in which the “value” of reward *R* after delay *D* is given by
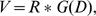
where *G*(*D*) is a discounting function that decreases with delay *D*. A steep rate of discounting results in impulsive choice, defined by an abnormally frequent choice of the more immediate reward [1], [2]. Serotonin (5-hydroxytryptamine; 5-HT), a major ascending neuromodulator, is thought to be involved in temporal discounting. Previous studies have reported that decreased serotonin levels resulted in impulsive choices [3]–[5] and that increased serotonin levels decreased impulsive choices [4], [6] (see Cardinal [7] for references, including those not conforming to this view). Lesions of specific parts of the cortico-basal ganglia loop also affect temporal discounting, whereas lesions in the orbitofrontal cortex promoted [8] or reduced [9] impulsive choices, and lesions in the dorsolateral part (core) of the nucleus accumbens resulted in impulsive choices [10], [11]. What neural network mechanism links serotonin to future reward evaluation and choice behavior? The following are our working hypotheses on the serotonergic regulation of delay discounting: 1) different sub-regions of topographically organized cortico-basal ganglia networks are specialized for reward prediction at different time scales, and 2) these sub-regions are differentially activated by ascending serotonergic systems [12]. In our previous brain imaging study [13], we demonstrated topographic maps of the time scales of reward prediction in the insular cortex and striatum in support of the first hypothesis. Here, we test the second hypothesis by combining dietary regulation of tryptophan, a precursor of serotonin, and functional magnetic resonance imaging (fMRI) during the performance of a choice task with variable delays.

## Results

### Behavioral results

We examined the effects of three different dietary tryptophan levels on the prediction of delayed rewards in a counter-balanced, placebo-controlled, double-blind, within-subject study (see Materials and Methods for details). Twelve subjects participated in experiments on three days with a minimum interval between experimental days of one week. Each day, a subject consumed one of three amino acid drinks: one containing a standard amount of tryptophan (control condition: 2.3 g per 100 g amino acid mixture), one containing excess tryptophan (trp+ condition: 10.3 g), and one without tryptophan (trp- condition: 0 g). Six hours after consumption and just before subjects performed behavioral tasks, the total plasma tryptophan levels of those under the trp- condition were significantly lower (*P*<0.0001; multiple comparison) than those under the control condition, while the levels of those under the trp+ condition were significantly higher (*P*<0.0001) (see Table S1). Based on previous studies of dietary tryptophan depletion [14]–[16] and loading [17], [18], we assume significant decreases and increases in central serotonin levels, respectively (see Text S1).

**Figure 1:**
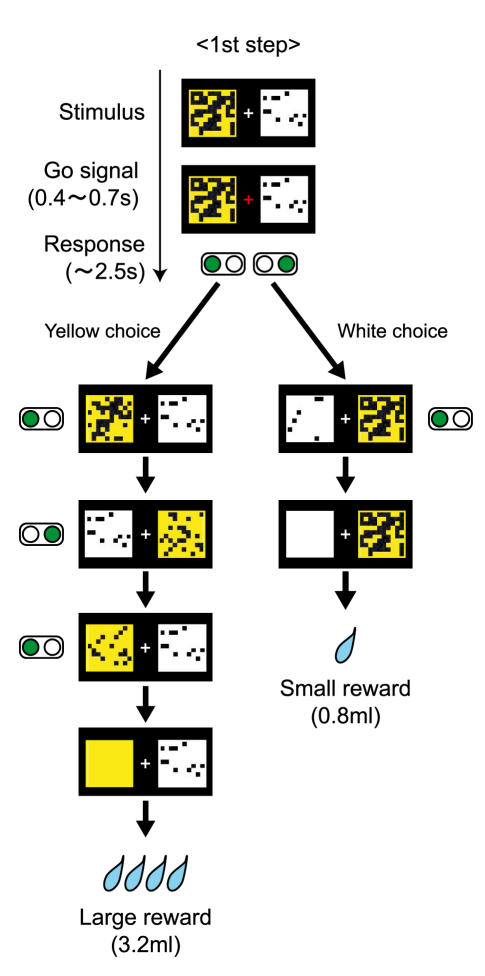
Experimental task. A subject was asked to select either a white or yellow square by pressing a button on the corresponding side. In the example shown here, if the subject chooses a white square at each step, a small amount of juice (0.8 ml) is delivered in two steps (2.5×2 = 5 sec). If the subject chooses a yellow square, four yellow choices (2.5×4 = 10 sec) must be repeated to obtain a larger amount of juice (3.2 ml). The position of the squares (left or right) was changed randomly at each step.

Under each tryptophan condition, all subjects performed a multi-step delayed reward choice task in an fMRI scanner (Fig. 1, see Materials and Methods for details). In this task, subjects chose between a white square indicating a small reward and a yellow square indicating a large reward. At the beginning of each trial, the white and yellow squares, occluded by variable numbers of black patches, were displayed side by side on a screen. After the fixation cross turned red, the subject selected either the white or yellow square by pressing a button on the corresponding side. The next set of squares was displayed 2.5 seconds after the previous one with a number of black patches removed from the selected square. When either square was completely exposed, a liquid reward was delivered to complete the trial. In the next trial, white and yellow squares were displayed with novel mosaic patterns. The initial number of black patches was randomly chosen from uniform distributions. The number of black patches removed at each step was also drawn randomly from uniform distributions, such that the delay until a small reward was usually shorter than the delay until a large reward. Thus, at the beginning of each trial, subjects needed to choose between the more immediate but small reward (white) and the more delayed but large reward (yellow) by comparing the number of black patches on the two squares. Since subjects needed to respond within 2.5 sec, they had to decide based on a visual impression of the darkness of the white and yellow squares, rather than explicitly counting the numbers of black patches and dividing them by the numbers of patches filled at each step.

Subjects performed eight sessions lasting three minutes each. Figure S1 shows an example of a subject's choice data in the *D_s_-D_l_* space (*D_s_*: delay for the small reward, *D_l_*: delay for the large reward): the subject predominantly chose large rewards when the delay to the large reward was relatively short (bottom right part of the *D_s_-D_l_* space), but preferred small rewards when the delay to the large reward was relatively long (top left part). No subject adopted an extreme strategy of choosing only small or large rewards in any session under any tryptophan condition (Fig. S2). There was no effect of tryptophan manipulation on the choice ratio of delayed reward and a number of other behavioral measures such as reaction times (see Text S1).

### Model-based regression analysis of fMRI signals and estimated V(t)

The levels of tryptophan significantly affected subjects' brain activity. We performed model-based fMRI data analyses based on an exponential discounting model (see Materials and Methods):
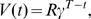
which was supported in our previous delay discounting choice task [19]. Here, value *V*(*t*) represents a discounted future reward *R* ( = 1 for white, 4 for yellow) acquired at time step *T* and evaluated at time step *t*. Because *V*(*t*) decreases exponentially with delay *D* = *T*-*t*, it grows exponentially as time *t* approaches *T* (see Fig. S3). Discount factor γ (0≤γ<1) controls the time scale of reward prediction; a smaller γ results in steeper discounting of future rewards, leading to short-term reward prediction. We assumed that subjects estimated the delay *D* = *T*-*t* until reward delivery from the number of black patches at each step, albeit with some uncertainty. Based on our hypothesis that different brain areas are involved in reward prediction at different time scales, we estimated *V*(*t*) with six different settings of γ (0.6, 0.7, 0.8, 0.9, 0.95, and 0.99) and used each of them as explanatory variables in a regression analysis.

**Figure 2:**
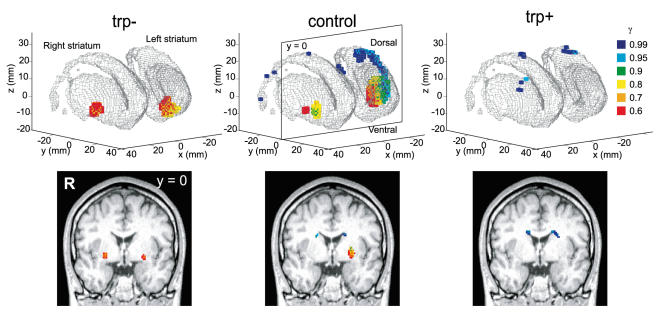
Regression analysis of BOLD signal by expected future reward with different discount rates. Voxels within the striatum (3D mesh surface) showing a significant correlation (*P*<0.001 in one sample t-test, uncorrected for multiple comparisons, *n* = 12 subjects) with *V*(*t*) at different settings of γ are shown with color codes (red: γ = 0.6, orange: 0.7, yellow: 0.8, green: 0.9, cyan: 0.95, blue: 0.99). Red to yellow coded voxels, correlated with reward prediction at shorter time scales, are predominantly located in the ventral part of the striatum (ventral putamen and nucleus accumbens), while the green to blue coded voxels, correlated with reward prediction at longer time scales, are located in the dorsal part of the striatum (dorsal putamen and caudate body).

We found that blood oxygen level-dependent (BOLD) signals in the striatum and the cortex correlated significantly with estimated *V*(*t*) (see Materials and Methods and Table S2). Under the control condition (Fig. 2, middle column), we found a significant correlation (*P*<0.001, uncorrected) of BOLD signals with *V*(*t*) at all γ values (0.6≤γ≤0.99) in the striatum (from the ventral part of the putamen to the body of the caudate nucleus), with a ventral to dorsal gradient (−4≤z≤28) from small to large γ. In the tryptophan depletion condition (trp-: Fig. 2, left column), we found a significant correlation (*P*<0.001, uncorrected) of BOLD signals with *V*(*t*) only at smaller γ values (0.6, 0.7, 0.8) in the ventral parts of the striatum (−12≤z≤−4). Conversely, in the tryptophan loading condition (trp+: Fig. 2, right column), we found a significant correlation (*P*<0.001, uncorrected) with *V*(*t*) only at larger γ values (0.9, 0.95, 0.99) in the dorsal parts of the striatum (16≤z≤28). These ventro-dorsal gradients could not reflect slice-timing effects within single scans as we sought a correlation with regressors varying across multiple scans, and also due to the fact that we acquired images by interleaved scanning (see Materials and Methods). We also performed model-based fMRI data analyses based on a hyperbolic discounting model (see Fig. S4). We found similar results as with the exponential model; a gradient map of discount rate in the control condition, significant correlation of *V*(*t*) with steeper discount rate only in the ventral putamen, and significant correlation of *V*(*t*) with slower discount rate only in the dorsal putamen and caudate nucleus.

**Figure 3:**
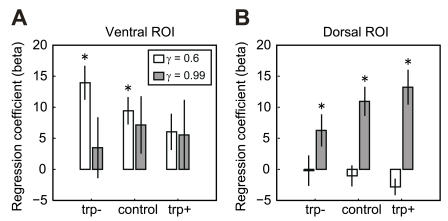
Effects of tryptophan levels on short- and long-term reward prediction in ventral and dorsal parts of the striatum. Bar plots show the regression coefficients (betas) of *V*(*t*) with γ = 0.6 (white bars) and 0.99 (gray bars) for the BOLD signals in the regions of interest (ROI) in the ventral (A) ((x, y, z) = (26, 0, −4) and (−26, 0, −8); see Table S2) and dorsal (B) ((x, y, z) = (24, 2, 22) and (−16, 2, 28)) parts of the striatum. Data shown are group averages (*n* = 12 subjects), and error bars represent standard errors. * significantly different from zero at *P*<0.05 in a two-tailed one sample t-test.

To quantify the differential modulation of the ventral and dorsal parts of the striatum by tryptophan levels, we set regions of interest (ROI) in the ventral and dorsal parts of the striatum (see Materials and Methods) and compared the regression coefficients (beta) of the BOLD signals with respect to reward prediction *V*(*t*) computed with small and large values of γ (0.6 and 0.99). While the activity of the ventral ROI (ventral putamen near the border of nucleus accumbens) correlated with *V*(*t*) with γ = 0.6 was strongest under the tryptophan depletion condition (Fig. 3A), the activity of the dorsal ROI (body of the caudate nucleus) correlated *V*(*t*) with γ = 0.99 was strongest under tryptophan loading (Fig. 3B). These results were confirmed by second-level analysis. For each subject, we checked the increasing or decreasing relationship between the betas and tryptophan levels by nonparametric analysis (Spearman's rank correlation coefficient). In the ventral ROI, the betas at small γ (γ = 0.6) showed a significantly decreasing relationship with tryptophan levels (*P* = 0.0215 in a one-tailed one-sample t-test), while the betas at large γ (γ = 0.99) showed no significance (*P* = 0.184). In the dorsal ROI, the betas at large γ showed a significantly increasing relationship with tryptophan levels (*P* = 0.010), while those at small γ showed no significance (*P* = 0.118).

To check for any effects of tryptophan levels on reward-unrelated brain activities or vascular responses, we investigated the event-related responses of the visual cortex at the beginning of each trial. We found no significant differences in the responses under different tryptophan conditions (*P* = 0.29, with anatomically defined ROI of V1 [20]), suggesting that the modulation found in the striatum was not due to a general effect of tryptophan levels on BOLD signals.

## Discussion

Our findings present the first evidence of an effect of the serotonergic system on localized brain activity related to reward prediction. Although we did not find significant differences in choice between immediate and delayed rewards at different tryptophan levels, as in previous human studies using dietary tryptophan depletion in healthy volunteers [21], we did observe significant differences in brain activities for reward prediction under different tryptophan levels. We found differential brain activities in the striatum with different tryptophan levels that could not be attributed to differences in motor components independent of subject choice preferences, because we did not find any significant effects of tryptophan levels on motor related measures, such as reaction time. Just as recent studies revealed differential genotypic effects by brain imaging [22], [23], the effects of neuropharmacological regulation may be more sensitively measured by local BOLD signal changes detected by fMRI than by behavioral output, which may be influenced by the entire brain. Thus, our present methods combining dietary tryptophan control and fMRI enabled us to detect effects of serotonin levels that would be difficult to detect by behavioral output alone.

Two main classes of model that characterize the shape of reward discounting have been proposed: exponential models [19], [24]–[26] and hyperbolic models [1], [27]. To analyze our brain imaging data, we used both models of both types, and we found a similar ventro-dorsal gradient of discount rates and modulation with tryptophan levels in the striatum for both models. These results show that our findings are not dependent on a particular choice of a mathematical model.

In the control tryptophan condition, we found a graded map of delay discount rate in the striatum; activities in the ventral portion were correlated with expected future rewards with steeper discounting, while those in the dorsal part were correlated with expected future rewards with slower discounting. This graded map of correlation was consistent with our previous study, which demonstrated that ventral cortico-basal ganglia loops are involved in short-term reward prediction and that dorsal loops are involved in long-term reward prediction [13]. Activation of the ventral part of the striatum by an immediate reward choice has also been recently reported [28].

One novel finding of our present study is that the parallel organization for reward prediction at different time scales in the striatum is under differential modulation by the central serotonergic system. We found a graded correlation from the ventral to dorsal parts of the striatum under control tryptophan conditions. In the ventral part, the correlation remained significant only in the tryptophan depletion condition. By contrast, in the dorsal part it remained significant only in the tryptophan loading condition. An ROI analysis confirmed that activity in the ventral part of the striatum, which is correlated with expected future rewards with steeper discounting, was enhanced under the tryptophan depletion condition, while activity of the dorsal part, which is correlated with expected future rewards with slower discounting, was enhanced under the tryptophan loading condition. These results support our hypothesis that different parts of the striatum are dedicated to reward prediction at different time scales and that they are differentially enhanced or suppressed by serotonergic modulation. Various subtypes of serotonin receptors with different affinities and intracellular effects are differentially distributed in the ventral and dorsal parts of the striatum [29], [30]. Such differential distributions of receptor subtypes could allow differential modulation of activities within the striatum under different serotonin levels. These mechanisms might be revealed by using particular receptor radioligands in a positron emission tomography (PET) experiment [31], [32].

## Materials and Methods

### Subjects

Twelve healthy right-handed males aged 22–25 years gave informed consent to participate in the experiment, which was conducted with the approval of the Review Board Ethics and Safety Committees for Functional Resonance Imaging Research of Advanced Telecommunication Research Institute International (ATR) and Hiroshima University. On the screening day, trained psychiatrists interviewed each volunteer and screened for previous psychiatric problems by using the Structured Clinical Interview for DSM-IV (SCID). We excluded volunteers with any previous psychiatric disorders, including mood or psychotic disorders, or substance abuse. Each volunteer had a physical that included blood and urine tests, a chest X-ray and an electrocardiogram. We excluded participants with health problems or those who disliked the isotonic drink used as the reward in the experiment.

### Tryptophan manipulation

Each subject participated for four days: one day for screening and task practice and three days for fMRI experiments under three different tryptophan conditions (depletion, trp-; loading, trp+; and control). Three days of experiments were scheduled over a minimal interval of one week to completely remove the effects of tryptophan dietary control induced in the preceding experiment. The experiment was of a counter-balanced, placebo-controlled, double-blind, within-subject design, in which a controller who was not an experimenter prepared three types of amino acid mixtures for each subject. To maximize dietary effect, subjects were instructed to consume the provided low-protein diet (less than a total of 35 g/day) 24 hours before the experiment and to fast overnight before each experimental day [18], [33], [34]. To motivate subjects for the liquid reward, water intake was restricted to 500 ml for 24 hours before each experiment.

On each experimental day, subjects consumed one of three amino acid mixtures (trp−, trp+, and control) and underwent two venipunctures to determine their total plasma tryptophan concentration, which is known to be correlated with CSF serotonin levels [14]–[18], [33]. The first blood samples were obtained before consumption of the amino acid mixture to confirm the tryptophan baseline, and the second blood samples were taken six hours after consumption to determine the effect of dietary manipulations on plasma tryptophan levels. A repeated-measures ANOVA showed a significant effect of dietary manipulations on total plasma tryptophan levels (*F*(2, 22) = 205.64, *P*<0.0001). After the second venipuncture, all subjects entered an fMRI scanner and performed the multi-step delayed reward choice task.

### Amino acid mixtures

We prepared amino acid mixtures consisting of the following quantities of 14 amino acids partially dissolved in 350 ml of water: L-tryptophan: 0 g (trp−), 10.3 g (trp+), 2.3 g (control), 5.5 g L-alanine, 4.9 g L-arginine, 3.2 g glycine, 3.2 g L-histidine, 8.0 g L-isoleucine, 13.5 g L-leucine, 11.0 g L-lysine monohydrochloride, 5.7 g L-phenylalanine, 12.2 g L-proline, 6.9 g L-serine, 6.5 g L-threonine, 6.9 g L-tyrosine, and 8.9 g L-valine. This aqueous suspension was flavored with 10 ml of chocolate syrup. In addition, 2.7 g of L-cysteine and 3.0 g of L-methionine were administered in a little water with each of the trp−, trp+ and control drinks, due to their unpalatability.

### Experimental task

Each experiment consisted of eight sessions lasting three minutes each. The initial numbers of black patches on white and yellow squares, *M_S_* and *M_L_* respectively, were *M_S_* = 18±9 and *M_L_* = 72±24 in all sessions. The numbers of patches added to white and yellow squares, *S_S_* and *S_L_* respectively, were varied between sessions so that subjects had to remain alert to adapt to the changes in settings: (*S_S_*, *S_L_*) = (6±2, 8±2) in four sessions, (6±2, 16±2) in two sessions, and (14±2, 8±2) and (14±2, 16±2) in one session each.

The expected delays to small and large rewards *D_S_* and *D_L_*, respectively, are given by:
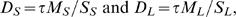
where *τ* is the time step (2.5 sec). Thus, the ranges of expected delays for the average values of *S_S_* and *S_L_* were: (*D_S_*, *D_L_*) = (3.75∼11.25 sec, 15∼30 sec) in four sessions, (3.75∼11.25 sec, 7.5∼15 sec) in two sessions, and (1.6∼4.8 sec, 15∼30 sec) and (1.6∼4.8 sec, 7.5∼15 sec) in one session each. However, because the experimental step was 2.5 sec, the actual delays were the above delays rounded to the next 2.5 sec increment.

As a liquid reward, an isotonic drink (Pocari Sweat, Otsuka Pharmaceutical Co., Ltd.) was delivered through a plastic tube from a computer-controlled pump (Harvard Apparatus, Inc., PHD 2000 Infusion) outside the MRI room. All subjects were told that they would receive a small drink (0.8 ml) after they completely filled the white square by selecting white squares; they would receive a larger drink (3.2 ml) when they filled the yellow square by choosing yellow squares.

At the beginning of each session, “baseline” blocks (25 sec) only showed a fixation cross. Each session lasted three minutes; the entire task took about 28 minutes. Although subjects could choose either square at any step, they did not usually reverse their choice; reversals accounted for about 8% of all choices, and trials with a reversed choice were excluded from analysis.

### Imaging data acquisition and preprocessing

A 1.5 Tesla scanner (Shimadzu-Marconi, MAGNEX ECLIPSE, Japan) was used to acquire both structural T1-weighted images and T2*-weighted echo planar images (TR = 2.5 s, TE = 55 ms, flip angle = 90°, 25 transverse slices, matrix = 64×64, FoV = 192 mm, thickness = 5 mm, slice gap = 0 mm) with BOLD contrast. Images were acquired with axial orientation in an interleave sequence in which even-numbered slices were first acquired from the top to the bottom of the brain, followed by the odd numbered slices. We used SPM2 (Wellcome Department of Imaging Neuroscience, Institute of Neurology, London, U.K.) for preprocessing and statistical analyses. The first five volumes of images were discarded to avoid T1 equilibrium effects. The images were realigned to the first image as a reference, spatially normalized with respect to the Montreal Neurological Institute (MNI) EPI template, and spatially smoothed with a Gaussian kernel (8 mm, full width at half maximum). We entered event-related regressors at the reward delivery event to capture head motion effects caused by swallowing the liquid reward.

### Model-based regression analysis

We estimated subjects' value *V*(*t*) at each step *t* as follows. By assuming an exponential discounting model, we estimated the discount factor γ for each subject in each condition (see Text S1). Based on the distribution of an estimated discount factor γ = 0.830±0.163 (mean±s.d., *n* = 34, excluding two samples of one subject in two conditions that had negative intercepts), we set γ for the value estimation as 0.6, 0.7, 0.8, 0.9, 0.95, and 0.99. We assumed that the subjects knew the mean number of patches filled at each step *s* and its range of variation Δ*s*. The range of possible steps *n* until the reward from the current number *M*(*t*) of black patches is
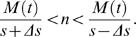
We defined estimated *V*(*t*) as the sum of *n*-step discounted value *R*γ*^n^* weighed by probability *P_n_*(*M*(*t*)) for all possible steps *n* until the reward:
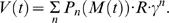
The probability of reaching the reward in *n* steps is given by
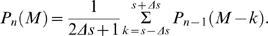


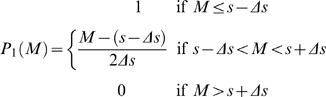



We used estimated *V*(*t*) at each γ as the explanatory variable in a general linear model (GLM) by multiplying the simple event regressor (δ-function) at the timing of the stimulus presentation of each step. To remove any effects of factors other than *V*(*t*), we concurrently used other variables in the regression, namely, reward *R* ( = 1 for white, 4 for yellow) at reward delivery timing and a boxcar function representing baseline blocks (25 sec) for eight sessions. All explanatory variables were convolved with a canonical hemodynamic response function (HRF). For each tryptophan condition, images of parameter estimates were created for each subject and entered into a second-level group analysis using a one sample t-test at a threshold of *P*<0.001, uncorrected for multiple comparisons (random effect analysis, *n* = 12). We repeated the same process for each γ and tryptophan condition.

To compare the results of regression analyses with six different values of γ, we used display software (multi_color: http://www.cns.atr.jp/multi_color/) that can overlay multiple activation maps in different colors on a single brain structure image. When a voxel is significantly activated in multiple values of γ, it is shown by a mosaic of multiple colors with apparent subdivision of the voxel.

### ROI analysis

We defined ROIs as clustered voxels in which we found significant (with a peak level threshold of *P*<0.001 (uncorrected) for the entire brain) correlations with *V*(*t*) at γ = 0.6 in trp- (368 mm^3^, union of two clusters peak at (x, y, z) = (26, 0, −4) and (−26, 0, −8); see Table S2), and γ = 0.99 in trp+ (88 mm^3^, union of two clusters peak at (x, y, z) = (24, 2, 22) and (−16, 2, 28)) within an anatomical ROI of the striatum, determined from normalized T1 images. We again used a GLM with averaged BOLD signal of all voxels within ROIs at the single-subject level and computed the value of the regression coefficient (beta) of *V*(*t*) averaged across subjects for each tryptophan condition. We used the MarsBaR toolbox for ROI analyses (http://marsbar.sourceforge.net/).

To check for an increased or decreased tendency between betas and tryptophan levels, we performed nonparametric analysis (Spearman's rank correlation coefficients) for each subject. In the group (second level) analysis, we found a significant normality of distribution of data for four groups, 1) γ = 0.6 in the ventral ROI, 2) γ = 0.99 in the ventral ROI, 3) γ = 0.6 in the dorsal ROI, and 4) γ = 0.99 in the dorsal ROI (*P*>0.05 in Kolmogorov-Smirnov test), and performed a one-sample t-test.

## Supporting Information

Text S1 Supplemental Methods, Results, Discussion and References.
(0.05 MB PDF)

Figure S1 A subject's choice (subject 11, control condition). Small and large reward choice on the D_s_-D_l_ space (D_s_: delay for the small reward, D_l_: delay for the large reward) and the indifference line, where the probabilities of the two choices are equal. Each asterisk (*) indicates where the subject chose a large reward with corresponding D_l_ against a small reward with corresponding D_s_, and each circle (o) indicates a choice of small reward against large reward.
(0.28 MB PDF)

Figure S2 Choice rate of large reward choices against small reward choices. The line plot shows the individual choice rate at each tryptophan level. A repeated measures ANOVA shows no effect of tryptophan levels on choice ratio of large rewards (F(2, 22) = 0.053, P = 0.948). All bar plots show mean across subjects and error bars indicate standard error.
(0.23 MB PDF)

Figure S3 Time course of estimated V(t) (subject 1, control condition). Each color corresponds to a value of γ used for calculating V(t) (corresponding to color code used in Fig. 2).
(0.38 MB PDF)

Figure S4 Regression analysis of BOLD signal by expected future reward using a hyperbolic model with different discount rates. We checked for a correlation between BOLD signal and hyperbolic discounted values (equation S2 in Text S1) with several different discount rates (k). We obtained very similar correlation maps with both exponential (Fig. 3 in the main text) and hyperbolic models (P<0.001 in a one sample t-test, uncorrected for multiple comparisons, n = 12 subjects).
(0.65 MB PDF)

Table S1 Total plasma tryptophan level before consumption and six hours after consumption.
(0.04 MB PDF)

Table S2 Voxels significantly correlated with estimated V (t) at each γ.
(0.07 MB PDF)
