# Supplementary material for: Serotonin Differentially Regulates Short- and Long-Term Prediction of Rewards in the Ventral and Dorsal Striatum
Source: PLoS One. 2007 Dec 19;2(12):e1333. doi: 10.1371/journal.pone.0001333 (PMC2129114; doi:10.1371/journal.pone.0001333)
Supplement: Text S1 — Supplemental Methods, Results, Discussion and References. (0.05 MB PDF) [file pone.0001333.s001.pdf]

Supporting Text S1

Tryptophan manipulation

We found that the concentration of total plasma tryptophan decreased by 92.5% (mean) under the depletion condition, six hours after treatment. In the previous study using the same amino acid mixture, the concentrations of total plasma tryptophan decreased by 82-94% (mean, 89%; six hours after treatment) and the concentration of cerebrospinal fluid (CFS) 5-HIAA, reflecting central serotonin levels, decreased by 24-40% (mean, 31%; 8.5 hours after treatment) [1]. In the tryptophan loading condition, we found that the concentration of total plasma tryptophan increased to about 12 times higher than baseline by six hours after treatment. In a previous study of tryptophan loading, the concentration of total plasma tryptophan quintupled (8 hours after treatment) and the concentration of CFS 5-HIAA increased significantly (8 hours after treatment) [2]. Based on these previous studies, we judge that our tryptophan depletion and loading treatments resulted in significant changes in the subjects' central serotonin levels.

Additional behavioral data analyses

A repeated-measures ANOVA showed no significant effects of tryptophan levels on the number of large reward choice ($F_{(2, 22)} = 0.053$, $P = 0.948$; Fig. S1) or on other measures such as reaction time ($F_{(2, 22)} = 0.586$, $P = 0.565$), total number of button presses ($F_{(2, 22)} = 2.79$, $P = 0.083$), number of trials in which subjects reversed their choices ($F_{(2, 22)} = 1.33$, $P = 0.286$), and total amount of rewards ($F_{(2, 22)} = 0.551$, $P = 0.584$).

Additional data analysis based on computational model of delay discounting

Exponential discounting of reward R at delay D,

$$V = R\ \gamma^{D}, \quad \text{(S1)}$$

is commonly used in artificial intelligence and economics because it enables an on-line learning algorithm and optimality under a constant rate of reward cancellation [3]. On the other hand, the hyperbolic discounting model,

$$V = R\ /\ (1 + kD), \quad \text{(S2)}$$

has often been used to explain animal choice behaviors [4,5].

A subject's choice behaviors can be characterized by plotting his/her small and large reward choices in the space spanned by the delay to small reward D_s and the delay to the large reward D_l [6]. While the exponential model predicts the slope of the "indifference line" equal to one (from $R_l\ \gamma^{Dl} = R_s\ \gamma^{Ds}$, we have $D_l = D_s + \log_{\gamma}(R_s/R_l)$), the hyperbolic model predicts the slope equal to $R_l/R_s = 4$ (from $R_l\ /\ (1 + kD_l) = R_s\ /\ (1 + kD_s)$).

Based on each subject's small and large reward choices at different points on D_s-D_l space, we performed logistic regression analyses of the probability P_l of a large reward (yellow) choice based on the following model:

$$P_l = 1/(1+\exp[\ -\ (\beta_l\ D_l + \beta_s\ D_s + \beta_0)]).$$

Each subject's choice indifference line was determined by setting $P_l = 0.5$, (*i.e.*, a line given by $D_l = -\ \beta_s/\beta_l\ D_s\ -\ \beta_0/\beta_l$) (see Fig. S1).

We found that the slopes of indifference lines β_s/β_l of the subjects were intermediate between hyperbolic (slope = 4) and exponential model (slope = 1) predictions (trp-: 2.82 ± 2.63 (mean ± s.d.), control: 2.49 ± 1.91, trp+: 2.72 ± 2.17). We did not find a significant effect of tryptophan levels on the slopes of the indifference lines ($F_{(2,22)} = 0.96$, $P = 0.909$). Subject discount factor γ can be estimated from the intercept of the indifference line in the D_s-D_l space given by $\log(R_s/R_l)/\log\gamma = \beta_0/\beta_l$. We did not find a significant effect of tryptophan levels on the intercept ($F_{(2,22)} = 1.10$, $P = 0.350$).

A note on striatal activities correlated with reward prediction and prediction error

In this study, we found that activities in the striatum correlated with reward prediction signals estimated by a computational model. This result is consistent with previous neural recording studies reporting reward expectation-related activities in the striatum [7-10]. However, a number of previous functional brain imaging studies have shown striatal activities to be correlated with reward prediction error [11-14]. The striatum receives both cortical input, representing sensory cues that allow reward prediction, and dopaminergic input from the substantia nigra, representing the reward prediction error signal for learning [15]. Thus, in an fMRI experiment, both reward prediction and prediction error signals can be detected as BOLD signals. In this study we found a correlation between BOLD signals and reward prediction, because the reward prediction error due to the uncertainty of the number of steps until receiving the reward was relatively small, compared with the steady build-up of reward expectation.

References

1. Carpenter LL, Anderson GM, Pelton GH, Gudin JA, Kirwin PD, et al. (1998) Tryptophan depletion during continuous CSF sampling in healthy human subjects. Neuropsychopharmacology 19: 26-35.

2. Young SN, Gauthier S (1981) Effect of tryptophan administration on tryptophan, 5-hydroxyindoleacetic acid and indoleacetic acid in human lumbar and cisternal cerebrospinal fluid. J Neurol Neurosurg Psychiatry 44: 323-328.

3. Sutton RS, Barto AG (1998) Reinforcement Learning.

4. Ainslie G (1975) Specious reward: a behavioral theory of impulsiveness and impulse control. Psychol Bull 82: 463-496.

5. Mazur JE (2001) Hyperbolic value addition and general models of animal choice. Psychol Rev 108: 96-112.

6. Mobini S, Chiang TJ, Al-Ruwaitea AS, Ho MY, Bradshaw CM, et al. (2000) Effect of central 5-hydroxytryptamine depletion on inter-temporal choice: a quantitative analysis. Psychopharmacology (Berl) 149: 313-318.

7. Kawagoe R, Takikawa Y, Hikosaka O (1998) Expectation of reward modulates cognitive signals in the basal ganglia. Nat Neurosci 1: 411-416.

8. Shidara M, Aigner TG, Richmond BJ (1998) Neuronal signals in the monkey ventral striatum related to progress through a predictable series of trials. J Neurosci 18: 2613-2625.

9. Tremblay L, Hollerman JR, Schultz W (1998) Modifications of reward expectation-related neuronal activity during learning in primate striatum. J

Neurophysiol 80: 964-977.

10. Samejima K, Ueda Y, Doya K, Kimura M (2005) Representation of action-specific reward values in the striatum. Science 310: 1337-1340.

11. McClure SM, Berns GS, Montague PR (2003) Temporal prediction errors in a passive learning task activate human striatum. Neuron 38: 339-346.

12. O'Doherty JP, Dayan P, Friston K, Critchley H, Dolan RJ (2003) Temporal difference models and reward-related learning in the human brain. Neuron 38: 329-337.

13. Seymour B, O'Doherty JP, Dayan P, Koltzenburg M, Jones AK, et al. (2004) Temporal difference models describe higher-order learning in humans. Nature 429: 664-667.

14. Tanaka SC, Doya K, Okada G, Ueda K, Okamoto Y, et al. (2004) Prediction of immediate and future rewards differentially recruits cortico-basal ganglia loops. Nat Neurosci 7: 887-893.

15. Schultz W, Dayan P, Montague PR (1997) A neural substrate of prediction and reward. Science 275: 1593-1599.
