# Supplementary figures and images for: Serotonin Differentially Regulates Short- and Long-Term Prediction of Rewards in the Ventral and Dorsal Striatum
Source: PLoS One. 2007 Dec 19;2(12):e1333. doi: 10.1371/journal.pone.0001333 (PMC2129114; doi:10.1371/journal.pone.0001333)

Supporting Figure S1

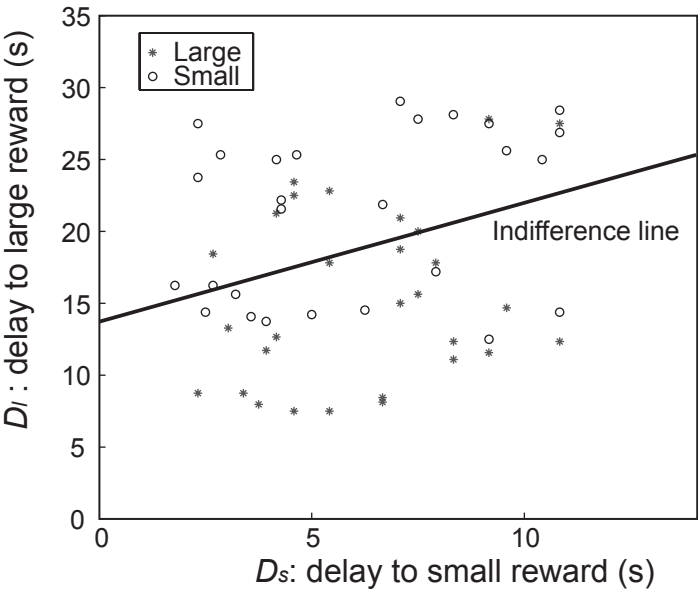

Supplement: Figure S1 — A subject's choice (subject 11, control condition). Small and large reward choice on the Ds-Dl space (Ds: delay for the small reward, Dl: delay for the large reward) and the indifference line, where the probabilities of the two choices are equal. Each asterisk (*) indicates where the subject chose a large reward with corresponding Dl against a small reward with corresponding Ds, and each circle (o) indicates a choice of small reward against large reward. (0.28 MB PDF) [file pone.0001333.s002.pdf]

Supporting Figure S2

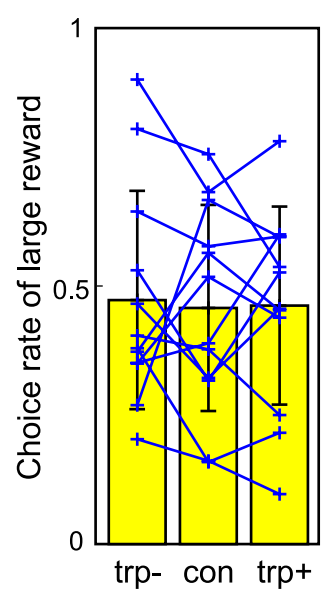

Supplement: Figure S2 — Choice rate of large reward choices against small reward choices. The line plot shows the individual choice rate at each tryptophan level. A repeated measures ANOVA shows no effect of tryptophan levels on choice ratio of large rewards (F(2, 22) = 0.053, P = 0.948). All bar plots show mean across subjects and error bars indicate standard error. (0.23 MB PDF) [file pone.0001333.s003.pdf]

# Supporting Figure S3

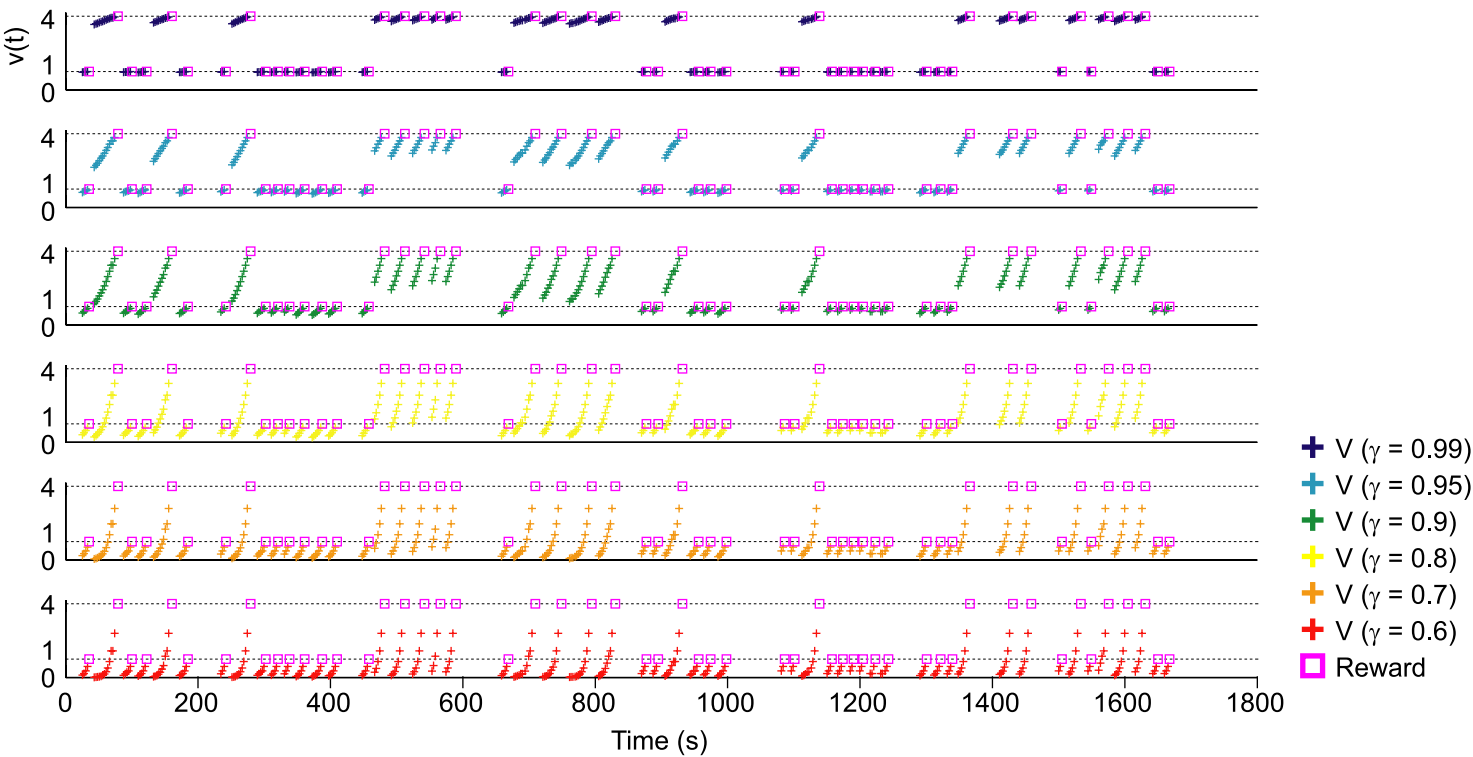

Supplement: Figure S3 — Time course of estimated V(t) (subject 1, control condition). Each color corresponds to a value of γ used for calculating V(t) (corresponding to color code used in Fig. 2). (0.38 MB PDF) [file pone.0001333.s004.pdf]

# Supporting Figure S4

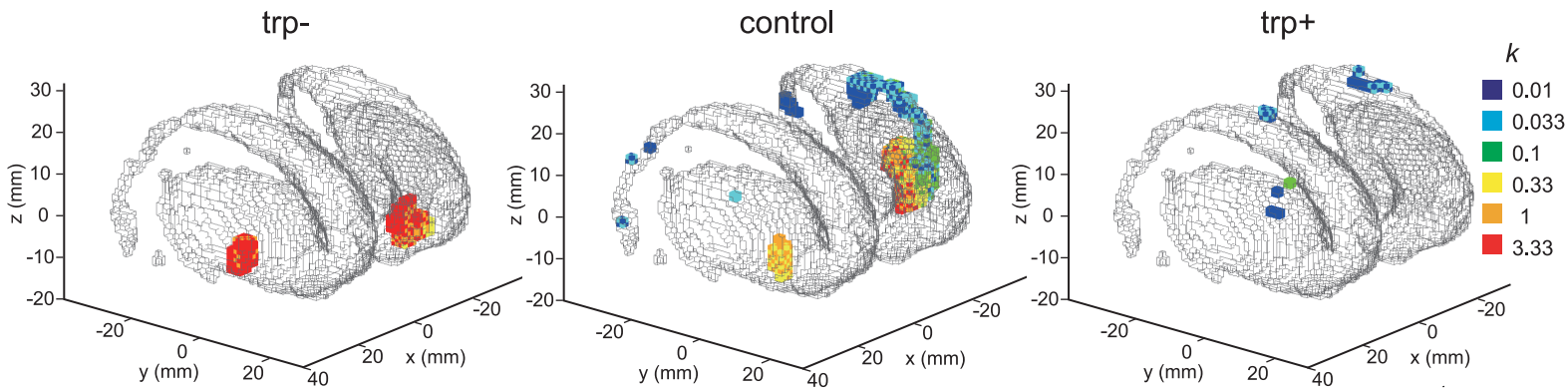

Supplement: Figure S4 — Regression analysis of BOLD signal by expected future reward using a hyperbolic model with different discount rates. We checked for a correlation between BOLD signal and hyperbolic discounted values (equation S2 in Text S1) with several different discount rates (k). We obtained very similar correlation maps with both exponential (Fig. 3 in the main text) and hyperbolic models (P<0.001 in a one sample t-test, uncorrected for multiple comparisons, n = 12 subjects). (0.65 MB PDF) [file pone.0001333.s005.pdf]
