# Supplementary material for: Serotonin Differentially Regulates Short- and Long-Term Prediction of Rewards in the Ventral and Dorsal Striatum
Source: PLoS One. 2007 Dec 19;2(12):e1333. doi: 10.1371/journal.pone.0001333 (PMC2129114; doi:10.1371/journal.pone.0001333)
Supplement: Table S1 — Total plasma tryptophan level before consumption and six hours after consumption. (0.04 MB PDF) [file pone.0001333.s006.pdf]

Supporting Table S1

Subject ID	trp- (mol/ml)		Control		trp+	
	Before	After	Before	After	Before	After
1	63.00	TR*	61.30	95.40	64.70	695.20
2	55.90	ND**	55.40	88.50	56.70	778.10
3	79.80	TR	78.70	170.20	71.30	872.30
4	48.70	ND	61.20	151.20	63.70	797.70
5	56.30	14.50	59.90	127.30	61.80	726.50
6	70.10	ND	90.40	159.70	79.50	740.90
7	68.10	ND	61.50	101.80	65.90	905.30
8	92.40	ND	75.90	162.50	81.90	626.20
9	70.80	8.50	69.60	329.30	54.90	889.90
10	58.60	ND	59.60	61.90	48.40	619.50
11	83.10	TR	70.70	103.20	68.50	761.00
12	58.60	ND	56.90	90.40	61.40	1004.90

Total plasma tryptophan level (mol/ml) before consumption (+0 h) and six hours after consumption (+6 h). *TR indicates more than 3.0 mol/ml and less than 5.9 mol/ml. **Not detectable (ND) indicates less than 2.9 mol/ml. In statistical tests, we used 2.9 mol/ml as ND and 5.9 mol/ml as TR.
