# Supplementary material for: Serotonin Differentially Regulates Short- and Long-Term Prediction of Rewards in the Ventral and Dorsal Striatum
Source: PLoS One. 2007 Dec 19;2(12):e1333. doi: 10.1371/journal.pone.0001333 (PMC2129114; doi:10.1371/journal.pone.0001333)
Supplement: Table S2 — Voxels significantly correlated with estimated V (t) at each γ. (0.07 MB PDF) [file pone.0001333.s007.pdf]

Supporting Table S2

	trp-			control			trp+		
	Area	T-value	Coordinates (x, y, z) Cluster size (mm^3)	Area	T-value	Coordinates (x, y, z) Cluster size (mm^3)	Area	T-value	Coordinates (x, y, z) Cluster size (mm^3)
γ = 0.6	Putamen	5.42	(26, 0, -4) 232	Putamen	5.72	(-26, 0, -2) 704			
	Putamen	4.92	(-26, 0, -8) 136	Putamen	4.17	(28,2,-4) 32			
	Nacc*	4.99	(-20, 10, -10) 240	Parietal cortex	5.86	(32, -58, 64) 1136			
γ = 0.7	Putamen	4.94	(26, 0, -4) 112	Putamen	5.84	(-24, 2, 2) 720			
	Putamen	4.35	(-26, 0, -6) 56	Putamen	4.67	(24,8,-2) 32			
	Nacc*	5.46	(-20,10,-10) 256	Parietal cortex	5.72	(32,-58,64) 1792			
γ = 0.8	Nacc*	6.16	(-20, 10, -12) 216	Putamen	5.71	(-24, 2, 4) 704			
				Putamen	5.28	(24, 8, -4) 152			
				Parietal cortex	6.01	(24,-72,58) 2488			
				Occipital cortex	8.59	(22, -98, 4) 584			
γ = 0.9				Putamen	5.02	(-24,2,4) 704	Putamen	5.38	(24,10,16) 56
				Putamen	4.41	(26, 8, -4) 40			
				Parietal cortex	6.81	(22,-72, 58) 2416			
				Occipital cortex	8.65	(-20, -98, 10) 848			
				Occipital cortex	10.04	(24, -98, 4) 1208			
γ = 0.95				Putamen	5.41	(-30, 6, 12) 1440	Putamen	4.59	(26,10,16) 112
				Putamen	4.05	(30, 0, 10) 8			
				Caudate	6.22	(24, 2, 24) 1280			
				Parietal cortex	6.7	(22, -52, 44) 2584			
				Occipital cortex	9.99	(-20,-98,10) 1336			
				Occipital cortex	8.33	(24, -96, 4) 2432			
γ = 0.99				Putamen	6.93	(-22,-8,12) 2184**	Caudate	6.45	(-16, 2, 28) 656
				Caudate	4.96	(-16, -2, 24) 2184**	Caudate	5.01	(24, 2, 22) 576
				Caudate	5.57	(26, 2, 28) 1512	Putamen	4.2	(28, 8, 8) 40
				Parietal cortex	6.54	(20, -52, 54) 2832			
				Occipital cortex	8.7	(-20, 96, 12) 1872			
				Occipital cortex	7.36	(24, -92, 4) 7344			
				Cerebellum	7.27	(38, -66, -40) 584			

Voxels significantly correlated with estimated V(t) at each γ ($P < 0.001$, uncorrected for multiple comparisons, $n = 12$). * Nacc: the most lateral part of nucleus accumbens. **These voxels are the different peaks at the large cluster.
